# Dihydroartemisinin induces apoptosis and inhibits proliferation, migration, and invasion in epithelial ovarian cancer via inhibition of the hedgehog signaling pathway

**DOI:** 10.1002/cam4.1827

**Published:** 2018-10-18

**Authors:** Yanmei Liu, Shujun Gao, Jie Zhu, Ya Zheng, Haiyan Zhang, Hong Sun

**Affiliations:** ^1^ Department of Gynecology, Obstetrics and Gynecology Hospital of Fudan University Shanghai China; ^2^ Shanghai Key Laboratory of Female Reproductive Endocrine Related Diseases Shanghai China; ^3^ The Diagnosis and Treatment Center of Cervical Disease, Obstetrics and Gynecology Hospital of Fudan University Shanghai China

**Keywords:** anticancer, dihydroartemisinin, hedgehog signaling pathway, ovarian cancer

## Abstract

Dihydroartemisinin (DHA), the primary of artemisinin extracted from the traditional Chinese medicine *Artemisia annua*, has been used in malaria treatment for a long time. Recently, many studies have indicated that, in addition to antimalarial effects, DHA also exhibits anticancer activity in certain types of neoplasms, including ovarian cancer. However, the precise anti‐ovarian cancer mechanism of DHA is still unclear. Abnormal activation of the hedgehog (Hh) pathway is closely related to tumorigenesis and progression of ovarian cancer. We performed this study to elucidate the effects of DHA on the biological behavior of ovarian cancer cells and to determine its effects on the Hh signaling pathway. CCK8 assays and flow cytometry were used to evaluate the effects of DHA on cell viability and apoptosis in both ovarian cancer cells and HOSEPICs (human ovarian surface epithelial cells) in response to DHA treatment. Transwell membrane chambers were used to analyze the effects of DHA on the migration and invasion of epithelial ovarian cancer cells following treatment with DHA. The impact of DHA on Hh signaling was analyzed by RT‐qPCR and Western blot. DHA significantly inhibited proliferation, migration, and invasion of ovarian cancer cells, and induced apoptosis in vitro. In contrast, DHA had few effects on cell proliferation and apoptosis in HOSEPICs. DHA inhibited the hedgehog signaling pathway. Furthermore, DHA inhibited purmorphamine (Hh signaling pathway agonist)‐induced cell proliferation, cell migration, and cell invasion and the inhibition of apoptosis. Importantly, DHA enhanced GANT61 (hedgehog signaling pathway inhibitor)‐induced apoptosis and the inhibition of cell viability, migratory capacity, and invasive ability. This study demonstrates that DHA inhibits cell viability, migration, and invasion, as well as induces apoptosis in epithelial ovarian cancer through suppression of the Hh signaling pathway.

## 1 INTRODUCTION

Ovarian cancer is the most fatal gynecologic tumor in women. An estimated 239 000 new cases of ovarian cancer and 152 000 deaths reportedly occur each year worldwide.^1^ Ovarian cancer is the leading cause of death in patients diagnosed with gynecological malignant tumors due to the difficulty of early diagnosis and resistance to chemotherapy. Epithelial ovarian cancer is the most predominant pathologic subtype of ovarian cancer, which makes up 90% of primary ovarian malignant tumors.^2^, ^3^ Cytoreductive surgery combined with platinum and paclitaxel‐based chemotherapy are standard treatment strategies for treating epithelial ovarian cancers; however, approximately 70% of patients relapse after initial treatment, often developing resistance to platinum‐based chemotherapy upon relapse.^4^ Ovarian cancer rapidly metastasizes in the short term and always develops resistance to chemotherapy. Hence, there are no satisfactory treatments for patients with late‐stage disease. The 5‐year survival rate for patients in late‐stage epithelial ovarian cancer is only 29%.^5^ Therefore, develop of a new, safe, and more effective drug with minimal side effects to control ovarian cancer cell’ s ability to invade and metastasize is urgent, to sequentially reduce mortality in patients.

Artemisinin is a sesquiterpene lactone isolated from a traditional Chinese medicine *Artemisia annua*.^6^ Many derivatives have been synthesized based on the structure of artemisinin, such as dihydroartemisinin (DHA), artesunate, and artemether, which have been used for the treatment of malaria for a long time.^7^ Recently, artemisinin and its derivatives have also been shown to exert antitumor effects in many tumors, such as lung cancer, breast cancer, prostate cancer, ovarian cancer, and so on.^8^, ^9^, ^10^, ^11^ DHA, the primary active products of artemisinin and its derivatives,^12^ exhibits the strongest anticancer effects among artemisinin and its derivatives.^13^ Furthermore, studies have shown that DHA has little effect on normal cells.^13^, ^14^ Numerous studies have shown that artemisinin inhibits the proliferation and metastasis of ovarian cancer cells and induces apoptosis.^11^, ^13^, ^15^ Therefore, DHA is a likely candidate for use in the treatment of ovarian cancer due to its minimal side effects.^16^

The Hh signaling pathway plays an important role in embryonic development by controlling cell proliferation and determining cell fate.^17^ In contrast, aberrant activation of hedgehog signaling is closely related to tumorigenesis and progression of many types of human cancer,^18^, ^19^, ^20^ including ovarian cancer.^18^ The sonic hedgehog signaling pathway consists of sonic hedgehog (Shh), patched (Ptch/Ptch1), smoothened, and GLI (glioma‐associated oncogene transcription factors).^21^ GLI1, a sign of aberrant activation of the Hh pathway, regulates expression of downstream target genes after activated, and its target genes are closely related to cancer cell survival, cell growth, and epithelial mesenchymal transition.^22^ In the absence of Hh ligand, Ptch inhibits the activity of smoothened (Smo). When Shh binds to Ptch, the inhibition of Smo by Ptch is relieved by Shh. Then, Smo transduces the signal by activating Gli‐1.^22^, ^23^ Aberrant activation of the Shh pathway is closely associated with tumorigenesis and progression of ovarian cancer. Many studies have confirmed that the sonic hedgehog pathway is aberrantly activated in ovarian cancer.^24^, ^25^, ^26^ Previous findings indicate that inhibiting Hh signaling by inhibiting Smo or GLI1 results in downregulation of GLI1, consequently inhibiting cell proliferation, cell migration, cell invasion, and inducing apoptosis in ovarian cancer.^25^, ^26^, ^27^ This highlights that inhibition of the Hh pathway may serve as a target for the treatment of ovarian cancer.

Although many studies have shown that DHA has potent antitumor effects in ovarian cancer, the exact anticancer mechanism of DHA is still not clear. Previous studies indicate that DHA inhibits the growth and progression of ovarian cancer by down‐regulating pFAK and MMP‐2;^11^ however, the underlying mechanisms through which DHA down‐regulates pFAK and MMP‐2 to inhibit epithelial ovarian cancer remain unclear. In 2003, a study reported that pFAK and MMP‐2 were downstream targeting genes of the hedgehog signaling pathway that induces cell migration and invasion through activation of FAK in ovarian cancer. Moreover, inhibition of the Hh pathway attenuated cell migration and invasion.^27^ Therefore, we hypothesized that DHA exerts anti‐ovarian cancer effects by inhibiting the hedgehog pathway.

In this study, our results reveal that DHA inhibits cell proliferation, migration, and invasion and induces apoptosis through inhibition of hedgehog signaling in ovarian cancer.

## 2 MATERIALS AND METHODS

### 2.1 Cell culture and agents

Ovarian cancer cell lines (SKOV3, SKOV3‐IP, HO8910, and HO8910‐PM) and human ovarian surface epithelial cells (HOSEPICs）were obtained from the Obstetrics and Gynecology Hospital of Fudan University. Cells were cultured in RPMI1640 medium supplemented with 10% fetal bovine serum and 1% penicillin and streptomycin. Cells were incubated in a humidified atmosphere consisting 5% CO2 at 37°C. DHA was purchased from Sigma Chemical Co. (St. Louis, MO). Purmorphamine and GANT61 were purchased from MedChemExpress (Monmouth Junction, NJ, USA). DMSO was used to dissolve DHA, purmorphamine, and GANT61. The final concentration of DMSO in all drug dilutions was <0.1%.

### 2.2 Cell proliferation assay

Cell viability was evaluated by CCK8 assay. Cells were seeded in 96‐well culture plates at a density of 5 × 10^3^ cells/well (SKOV3, SKOV3‐IP and HOSEPIC) and 3 × 10^3^ cells/well（HO8910 and HO8910‐PM）. After starvation with FBS‐free medium for 10 hours, cells were treated with different concentrations of DHA, 1.5 μM of purmorphamine, and 20 μM of GANT‐61 for 24， 48, and 72 hours each. Controls were treated with DMSO vehicle alone. Upon completion of incubation time points, cells were treated with 10 μL CCK‐8 reagent (MedChemExpress) and cultured at 37°C for approximately 45 minutes. Absorbance at 450 nm was measured to evaluate cell viability.

### 2.3 Apoptosis assay

BD Pharmingen Annexin V‐FITC Apoptosis Detection Kit I (BD Biosciences, Franklin Lakes, NJ, USA) was used to measure apoptosis. Cells were seeded in 6‐well plates at a density of 3.5 × 10^5^ cells/well and treated with various concentrations of DHA (20, 40, 80, and 160 μM), 1.5 μM purmorphamine, and 20 μM of GANT‐61. After incubating for 48 hours, cells were washed with ice‐cold PBS and resuspended in annexin‐binding buffer, followed by treatment with Annexin V‐FITC and PI reagent for 15 minutes in the dark. Apoptosis was measured by flow cytometry.

### 2.4 Migration assay

Cell migration was assessed using an in vitro transwell chamber migration assay. Cells were seeded into the upper chambers of a transwell insert (Corning, New York, NY, USA). Cells were treated with DHA (40 μM) and DMSO for experimental and control groups, respectively, for 24 hours. The lower chamber contained 600 μL RPMI1640 medium supplemented with 20% FBS. Nonmigrating cells on the top of the polycarbonate membrane were removed using cotton swabs. Migrated cells on the bottom of the membrane were fixed with 1% paraformaldehyde for 30 minutes and stained with 2% crystal violet for 30 minutes. Migration images were acquired using an inverted microscope.

### 2.5 Invasion assay

An in vitro transwell chamber invasion assay was used to detect the invasive ability of cells. Cells were starved for approximately 14 hours in FBS‐free culture medium. Matrigel was diluted 1:6 in FBS‐free RPMI1640, and 50 μL diluted Matrigel was spread into the upper chamber of the transwell overnight. Cells were seeded into the upper chamber of an 8.0‐µm polycarbonate membrane transwell at a density of 2.5 × 10^4^ cells/well for SKOV3 and SKOV3‐IP cells and 2 × 10^4^ cells/well for HO8910 and HO8910‐PM cells. The experimental groups were treated with 40 μM DHA, and control groups were treated with DMSO for 48 hours. The lower chamber contained 600 μL of RPMI1640 medium with 20% FBS. Non‐invading cells and Matrigel on top of the polycarbonate membrane were removed using cotton swabs. Invading cells on the bottom of the membrane were fixed with 1% paraformaldehyde for 30 minutes and stained with 1% crystal violet. Invasion images were acquired using an inverted microscope.

### 2.6 Real‐time quantitative PCR

Total cellular RNA was isolated from ovarian cancer cells using Trizol (Invitrogen, Carlsbad, CA, USA). HiScriptQ RT SuperMix for qPCR (Vazyme, Nanjing, China) was used to synthesize cDNA as directed by the manufacturer. qRT‐PCR was performed using SYBR Color Qpcr Master Mix (Vazyme) as directed by the manufacturer. Primer sequences are shown in Table 1. Relative mRNA expression levels of Shh, Ptch1, Smo, and GLI1 were analyzed by the 2^−ΔΔCt^ method. β‐actin was chosen as an internal control. qRT‐PCR experiments were repeated three times.

**Table 1:** The primer sequences for qRT‐PCR

Gene	Forward primers	Reverse primers
Shh	GTGATGAACCAGTGGCCAGG	GCCCTCGTAGTGCAGAGACT
Ptch1	TAATGACTCCCAAGCAAATG	GACACTCTGATGAACCAC C
Smo	GATCCCGTCTTCCAGAGAAC	CCACAGTCC TGCTGCTTTGA
Gli‐1	GGACCCAACTTGCCCAATCA	GGACCCAACTTGCCCAATCA
β‐actin	AAGGTGACAGCAGTCGGTT	AAGGTGACAGCAGTCGGTT

### 2.7 Western blot analysis

RIPA containing PMSF and phosphotransferase inhibitors (Beyotime Technology, Jiangsu, China) was used for protein extraction. Protein concentration was measured using a BCA protein assay kit (Beyotime Institute of Biotechnology). Equivalent protein concentrations were separated using SDS‐PAGE (12. 5%) and transferred onto PVDF membranes, which were subsequently blocked with 5% nonfat milk and incubated with relevant primary antibodies at 4°C overnight. Horseradish peroxidase‐conjugated secondary antibodies were used to detect primary antibodies. The ECL detection system (Millipore, Billerica, MA, USA) was used to image protein bands. Primary antibodies included anti‐β‐actin (1:1,000, ab8226, Abcam, Cambridge, MA, USA), anti‐Shh (1:1,000，ab53281，Abcam), anti‐Gli‐1 (1:1,000, ab134906, Abcam), anti‐Ptch1 (1:500, ab53715, Abcam), and anti‐Smo (1:1,000, ab72130, Abcam).

### 2.8 Data analysis

The results are expressed as the means ± SEM of three independent experiments. Student's *t* test or one‐way ANOVA was used to analyze significant differences.

A *P* value <0.05 was considered statistically significant.

## 3 RESULTS

### 3.1 DHA inhibits the proliferation of ovarian cancer cells

**Figure 1:**
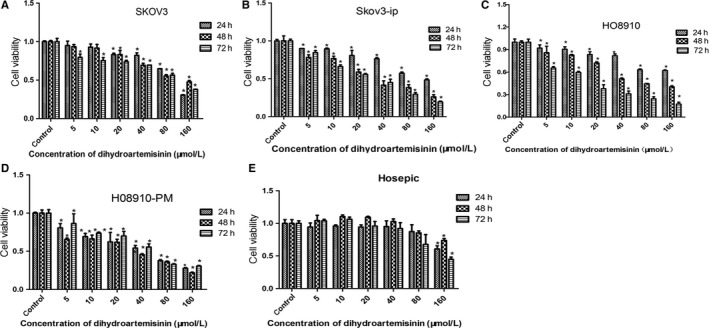
Dihydroartemisinin (DHA) inhibits cell viability of human ovarian cancer cell lines (SKOV3, SKOV3‐IP, HO8910, and HO8910‐PM). Cells were treated with 5‐160 μM DHA, and controls were treated with DMSO. Cell viability was assessed using CCK8 assay after treatment with different doses of DHA or DMSO for 24, 48, and 72 h. Data are expressed as the mean ± SEM of three independent experiments. *P < 0.05, **P < 0.01 and ***P < 0.001, compared to controls

Cell viability was assessed by CCK8 assay. After 24, 48, and 72 hours of DHA treatment, a dose‐dependent decrease in cell viability was observed in SKOV3, SKOV3‐IP, HO8910, and HO8910‐PM cells in response to increasing concentrations of DHA (ranging from 5 to 160 μM) in vitro (Figure 1A‐D). In HO8910 cells, we observed that DHA inhibited cell proliferation in a time‐dependent manner. After treatment with DHA for 72 hours, cell viability was lowest compared to treatment with DHA at 24 and 48 hours. However, DHA did not inhibit cell viability of SKOV3, SKOV3‐IP, or HO8910‐PM cells in a time‐dependent manner. Furthermore, different kinds of ovarian cancer cells exhibited differential sensitivity to DHA. SKOV3‐IP and HO8910‐PM cells were more sensitive to DHA than SKOV3 and HO8910 cells. Approximately, 20 μM of DHA resulted in approximately 50% cell death in SKOV3‐IP and HO8910‐PM cells after 48 hours DHA treatment, while 44 μM DHA was required to induce approximately 50% growth inhibition in HO8910 cells after 48 hours treatment. However, in SKOV3, 89 μM DHA was required to affect approximately 50% cell death. DHA had little effect on cell viability of HOSEPICs, and only 160 μM DHA managed to decrease cell viability in this line (Figure 1E).

### 3.2 DHA induces apoptosis in ovarian cancer cells

**Figure 2:**
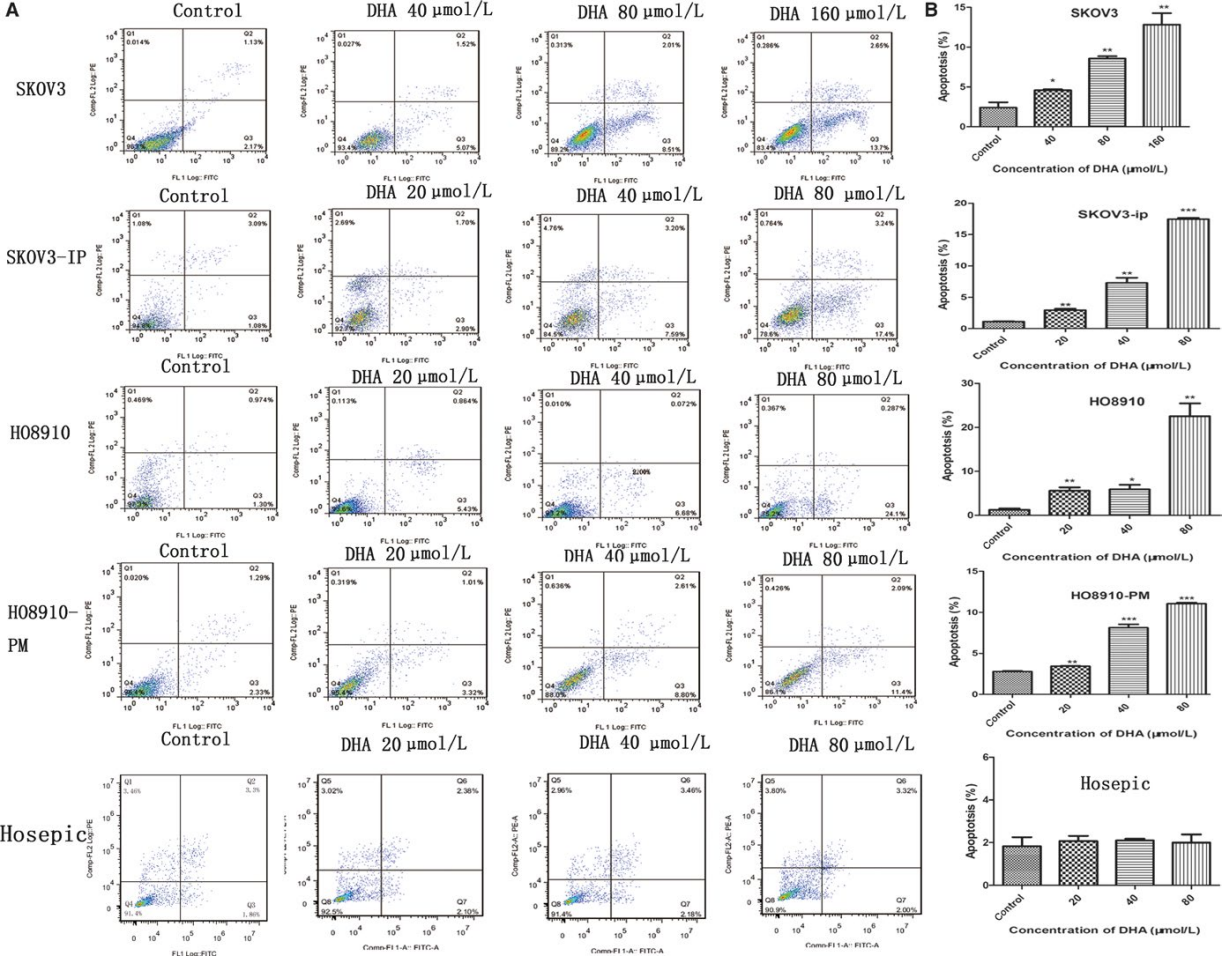
DHA induces apoptosis in ovarian cancer. The Annexin V‐FITC Apoptosis Detection Kit I and flow cytometry were used to measure apoptosis in SKOV3, SKOV3‐IP, HO8910, HO8910‐PM, and HOSEPIC cells following treatment with different doses of DHA for 48 h. The control group was treated with DMSO. Q3 represents early apoptosis. Data are expressed as the mean ± SEM of three separate experiments. *P < 0.05, **P < 0.01 and ***P < 0.001, compared to controls

Apoptosis was analyzed using the Annexin V‐FITC Apoptosis Detection Kit I and flow cytometry. SKOV3, SKOV3‐IP, HO8910, and HO8910‐PM cells were treated with different concentration of DHA according to the IC50. As a result, the proportion of early apoptotic cells increased significantly in a dose‐dependent manner in all ovarian cancer cells following DHA treatment for 48 hours. (Figure 2). In SKOV3 cells, the percentage of early apoptotic cells increased from 2.4% (DMSO treated) to 4.6%, 8.6%, and 12.8% when cells were treated with 40, 80, and 160 μM DHA, respectively. In SKOV3‐IP cells, early apoptotic cells increased from 1.11% (DMSO treated) to 2.9%, 7.3%, and 17.4% when cells were treated with 20, 40, and 80 μM DHA, respectively. Similarly, the apoptotic index increased with increasing concentrations of DHA in HO8910 and HO8910‐PM cells. However, 20‐80 μM of DHA had no effect on apoptosis of HOSEPICs compared to controls, consistent with cell proliferation experiments.

### 3.3 DHA inhibits migration of ovarian cancer

**Figure 3:**
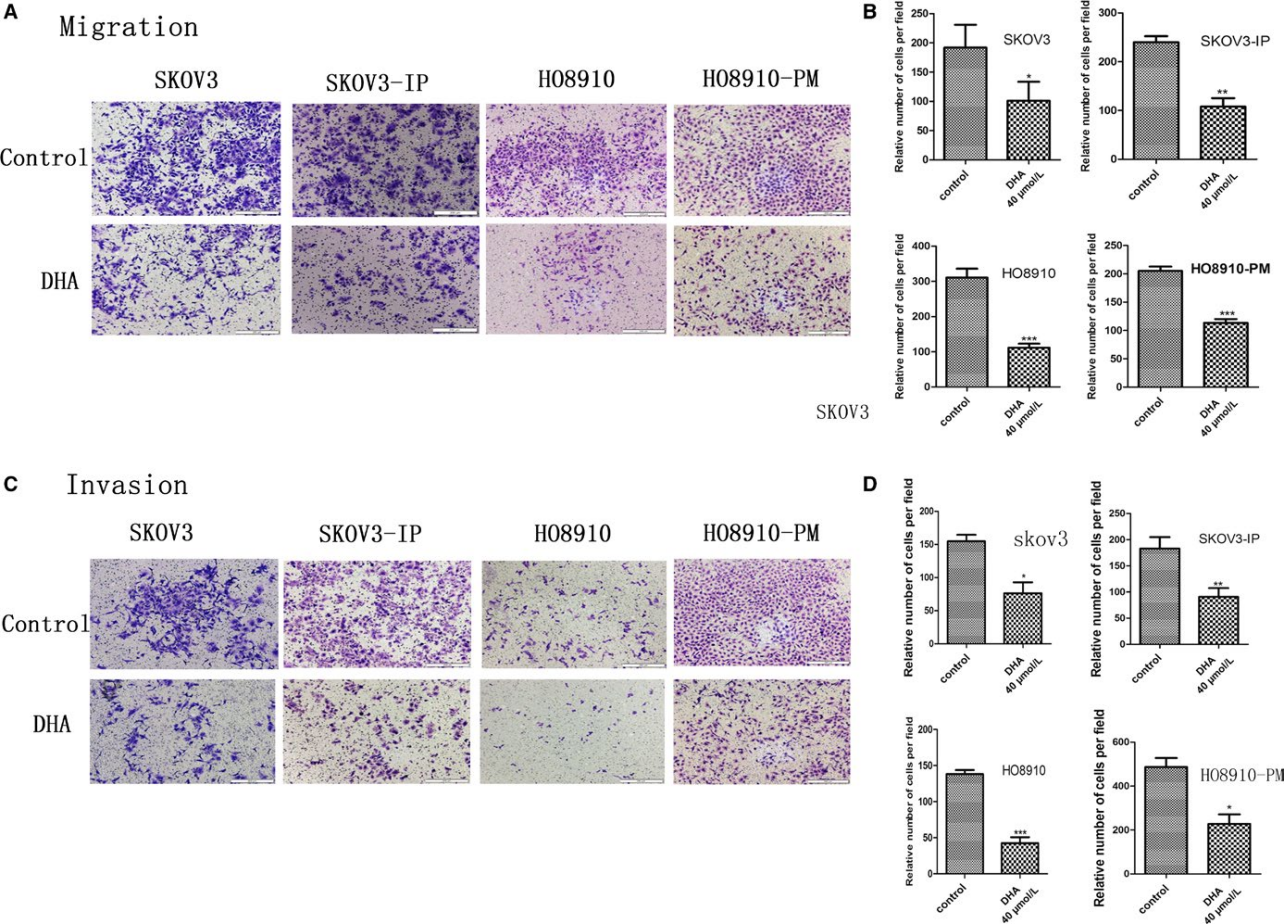
DHA inhibits migration and invasion of ovarian cancer cells. An in vitro transwell chamber migration assay and Matrigel invasion assay were used to evaluate the migratory and invasive capabilities of ovarian cancer cells following treatment with 40 μM DHA or DMSO for 24 and 48 h, respectively. A, Images of migrated cells, which were recorded using an Olympus microscope (10×). C, Images of invaded cells, which were recorded using an Olympus microscope (10×). B and D, Average number of migrated and invaded cells from five randomly selected fields. Data represent the mean ± SEM of three independent experiments. *P < 0.05, **P < 0.01 and ***P < 0.001, compared to controls

To investigate the effects of DHA on SKOV3, SKOV3‐IP, HO8910, and HO8910‐PM cell migratory potential, an in vitro transwell chamber migration assay was used to detect cell migration. We selected 40 μM DHA to treat ovarian cancer cells for 24 hours, which significantly inhibited the migratory capability of ovarian cancer cells compared to control groups (Figure 3A,B). The number of migratory DHA‐treated SKOV3, SKOV3‐IP, HO8910, and HO8910‐PM cells was approximately 49%, 45%, 36%, and 55%, respectively, that of the control group. Our data indicate that DHA significantly suppresses the migration ability of ovarian cancer cells.

### 3.4 DHA inhibits invasion of ovarian cancer cells

Invasion is a very important biological characteristic of cancer cells. The invasion assay was conducted using a Matrigel‐coated transwell chamber assay. Our data revealed that treatment with 40 μM DHA for 48 hours significantly suppressed the invasion of ovarian cancer cells. The number of invading DHA‐treated SKOV3, SKOV3‐IP, HO8910, and HO8910‐PM cells was approximately 69.7%, 48.9%, 69.2%, and 53.1%, respectively, that of the control group (Figure 3C,D).

### 3.5 DHA inhibits the hedgehog signaling pathway in ovarian cancer

**Figure 4:**
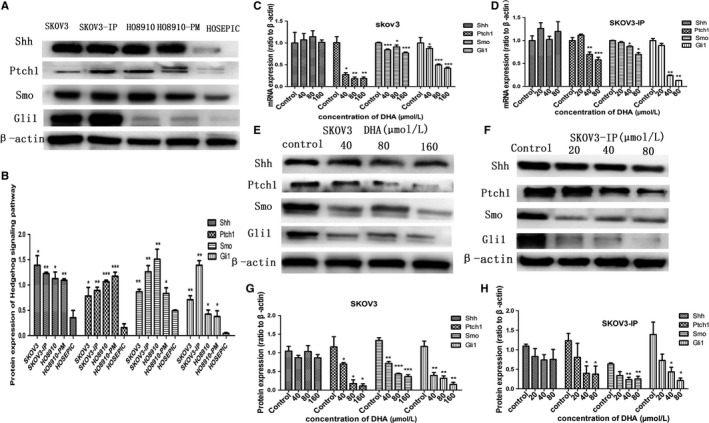
DHA inhibits hedgehog signaling in ovarian cancer. SKOV3 cells were treated with DHA at concentrations of 40, 80, and160 µM for 48 h, and SKOV3‐IP cells were treated with DHA at concentrations of 20, 40, and 80 µM for 48 h, while controls were treated with DMSO. A and B, Protein expression of the hedgehog pathway components in ovarian cancer cell lines and HOSEPICs. C and D, RT‐qPCR analysis of Hh pathway component mRNA expression in SKOV3 and SKOV3‐IP cells following treatment with DHA (20‐160 µM) for 48 h, and results were normalized to β‐actin to correct for loading. E and F, Protein expression of Hh pathway components in SKOV3 and SKOV3‐IP cells following treatment with DHA for 48 h measured by Western blot; β‐actin served as an internal control. G and H, Bar graphs represent protein expression of Hh pathway components. All experiments were repeated three times. *P < 0.05, **P < 0.01 and ***P < 0.001, vs the control group

Recently, many studies have confirmed that the Hh pathway is activated in ovarian cancer and that aberrantly activated Hh signaling promotes tumorigenesis and development of ovarian cancer. Our study revealed elevated protein expression of Shh, Ptch1, Smo, and Gli‐1 in four ovarian cancer cells compared to HOSEPICs (Figure 4A,B), indicating that Hh signaling is activated in ovarian cancer. To elucidate whether DHA exerted anti‐ovarian cancer effects via inhibiting the hedgehog pathway, we explored the effects of DHA on expression of Shh, Ptch1, Smo, and GLI1 following treatment with DHA for 48 hours. SKOV3 and SKOV3‐IP cell lines were used for further study due to their stable protein expression of Gli‐1 based on results of preliminary experiments. SKOV3 cells were treated with DHA (40‐160 μM) for 48 hours, SKOV3‐IP cells with DHA (20‐80 μM) for 48 hours, meanwhile, and control cells with DMSO. The results showed that mRNA expression of Ptch1, Smo, and GLI1 decreased significantly with increasing concentrations of DHA; however, there was no significant difference in Shh expression between experiment and control groups (Figure 4C,D). In accordance with mRNA, our studies showed that DHA also inhibited Ptch1, Smo, and GLI1 protein levels, whereas, DHA had no effect on the Shh protein level (Figure 4E‐H). These results demonstrate that DHA inhibits the hedgehog (Hh) pathway potentially through inhibition of Smo expression.

### 3.6 Hh signaling pathway is involved in the DHA‐induced inhibition of cell proliferation

**Figure 5:**
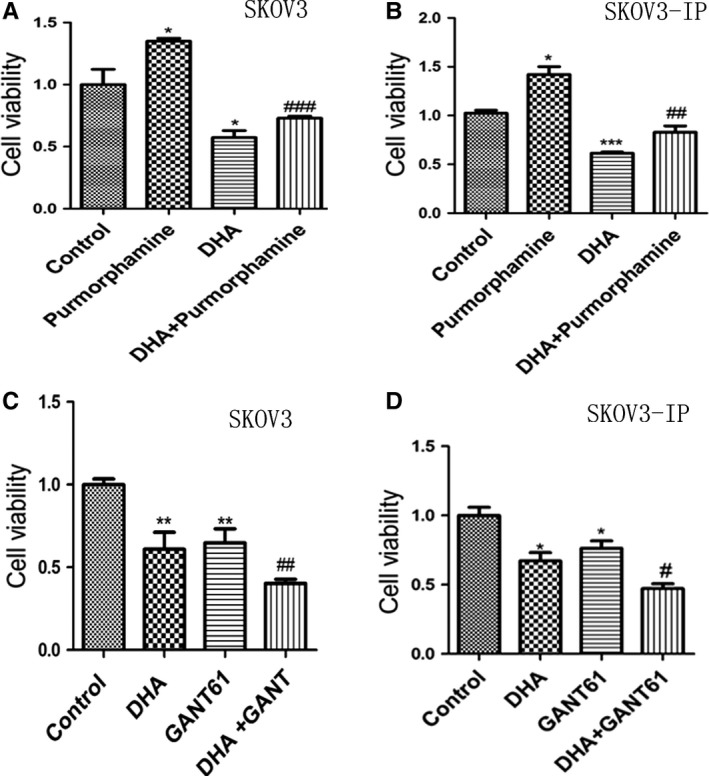
The Hh signaling pathway is involved in DHA‐induced inhibition of cell proliferation. A and B, CCK8 assay of SKOV3 and SKOV3‐IP cells was conducted following treatment with purmorphamine, DHA or a combination of DHA and purmorphamine for 48 h. C and D, SKOV3 and SKOV3‐IP cells were treated with GANT61, DHA, or a combination of DHA and GATN61 for 48 h, while controls were treated with DMSO. CCK8 assay was used to analyze cell viability of SKOV3 and SKOV3‐IP cells. *P < 0.05, **P < 0.01 and ***P < 0.001, vs the control group. ^#^ *P* < 0.05, ^##^ *P* < 0.01 and ^###^ *P* < 0.001, vs the purmorphamine group GANT61 groups

To further examine whether DHA induced the inhibition of cell viability of ovarian cancer through inhibition of the Hh pathway, we treated cells with purmorphamine (a smoothened agonist, and specific activator of the Hh signaling pathway), DHA, or a combination of purmorphamine and DHA for 48 hours. We also treated cells with GANT61 (a Gli‐1/Gli2 inhibitor), DHA, or combination of GANT61 and DHA for 48 hours, while control cells were treated with DMSO. The effect of purmorphamine, GANT61, DHA, purmorphamine combined with DHA, and GANT61 combined with DHA on the proliferation of SKOV3 and SKOV3‐IP cells was assessed by CCK8 assay. Treatment with 1.5 μM of purmorphamine significantly increased SKOV3 and SKOV3‐IP cell viability. Consistent with previous results of cell proliferation assay, 80 μM of DHA significantly inhibited proliferation of SKOV3 cells, and 40 μM of DHA significantly decreased cell viability in SKOV3‐IP cells. Combination of DHA and purmorphamine inhibited purmorphamine‐induced acceleration of cell viability in both SKOV3 and SKOV3‐IP cells (Figure 5A,B). Treatment with 20 μM GANT61 decreased cell viability of SKOV3 and SKOV3‐IP cells to 65% and 63%, respectively, and treatment with a combination of DHA and GANT61 significantly augmented GANT61‐induced inhibition of cell proliferation (Figure 5C,D).

### 3.7 Hh signaling pathway is involved in DHA‐induced apoptosis

**Figure 6:**
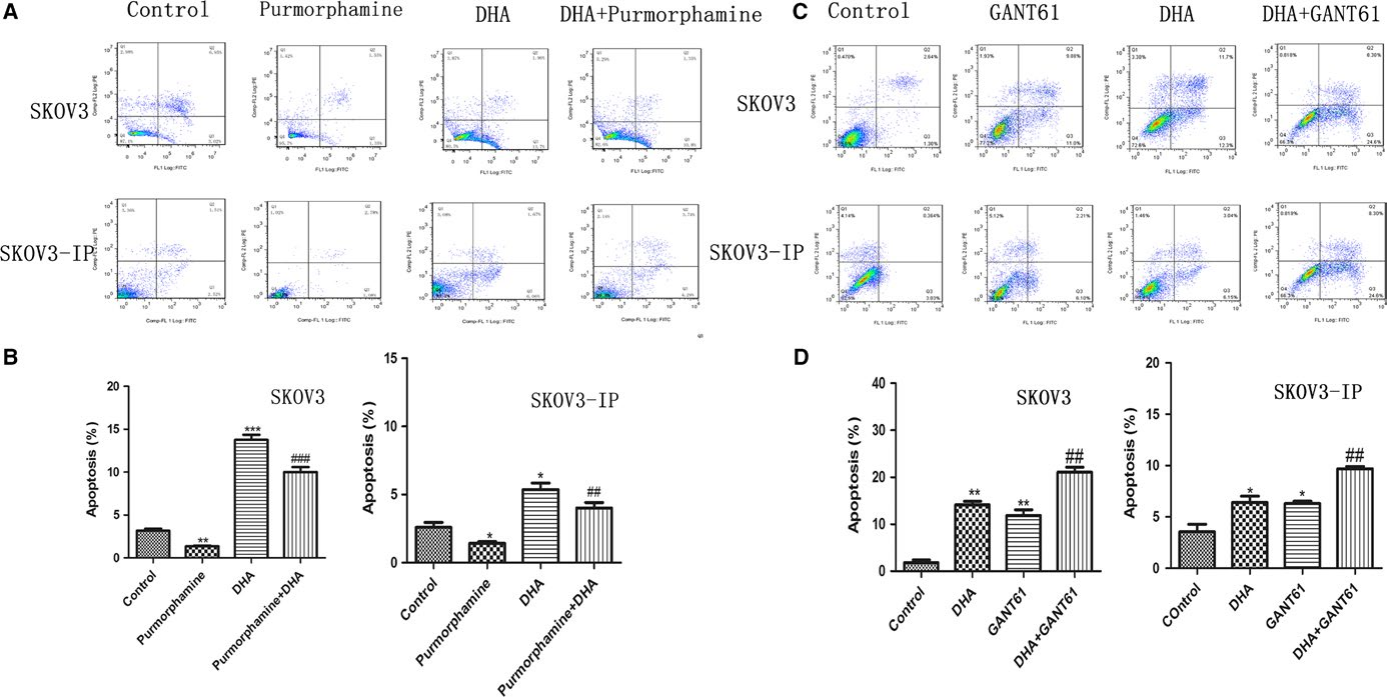
The Hh signaling pathway is involved in DHA‐induced apoptosis. A and B, SKOV3 and SKOV3‐IP cells were treated with 1.5 µM of purmorphamine, DHA, or combined DHA and purmorphamine for 48 h. C and D, SKOV3 and SKOV3‐IP cells were treated with 20 µM GANT61, DHA, or combined DHA and GANT61 for 48 h. The concentration of DHA in SKOV3 and SKOV3‐IP was 80 and 40 µM, respectively, and the control group was treated with DMSO. Apoptosis was analyzed by flow cytometry. All experiments were repeated three times. *P < 0.05 and **P < 0.01, vs the control group. ^#^ *P* < 0.05, ^##^ *P* < 0.01 and ^###^ *P* < 0.001, vs the purmorphamine and GANT61 groups

To investigate the relationship between apoptosis induced by DHA and the Hh pathway, we treated SKOV3 and SKOV3‐IP cells with purmorphamine, DHA alone, or combined DHA and purmorphamine for 48 hours. We found that 1.5 μM of purmorphamine inhibited apoptosis in both SKOV3 and SKOV3‐IP cells (Figure 6A,B). DHA reversed the effect of purmorphamine‐induced inhibition of apoptosis. The percentage of cells in early apoptosis increased from 1.35% (purmorphamine‐treated group) to 10% (combination of DHA and purmorphamine‐treated group) in SKOV3, and from 1.4% (purmorphamine‐treated group) to 4%(combined of DHA and purmorphamine‐treated group) in SKOV3‐IP cells. We also treated SKOV3 and SKOV3‐IP cells with DHA alone, GANT61 alone, or combined DHA and GANT61 for 48 hours. As demonstrated in Figure 6C,D, 20 μM of GANT61 increased apoptosis more than fivefold in SKOV3 cells, and increased apoptosis by about twofold in SKOV3‐IP cells compared to the control group. Nevertheless, apoptosis in the combined treatment group increased from 11.8% (GANT61‐treated group) to 21% (combined DHA and GANT61‐treated group) in SKOV3 cells, and from 6. 3% (GANT61‐treated group) to 9.7% (combined DHA and GANT61‐treated group) in SKOV3‐IP cells. These results indicate that DHA inhibits purmorphamine‐induced inhibition of apoptosis and enhances GANT61‐induced apoptosis. Therefore, the Hh pathway may be associated with DHA‐induced apoptosis.

### 3.8 DHA suppresses migration and invasion of ovarian cancer cells through inhibition of the Hh pathway

**Figure 7:**
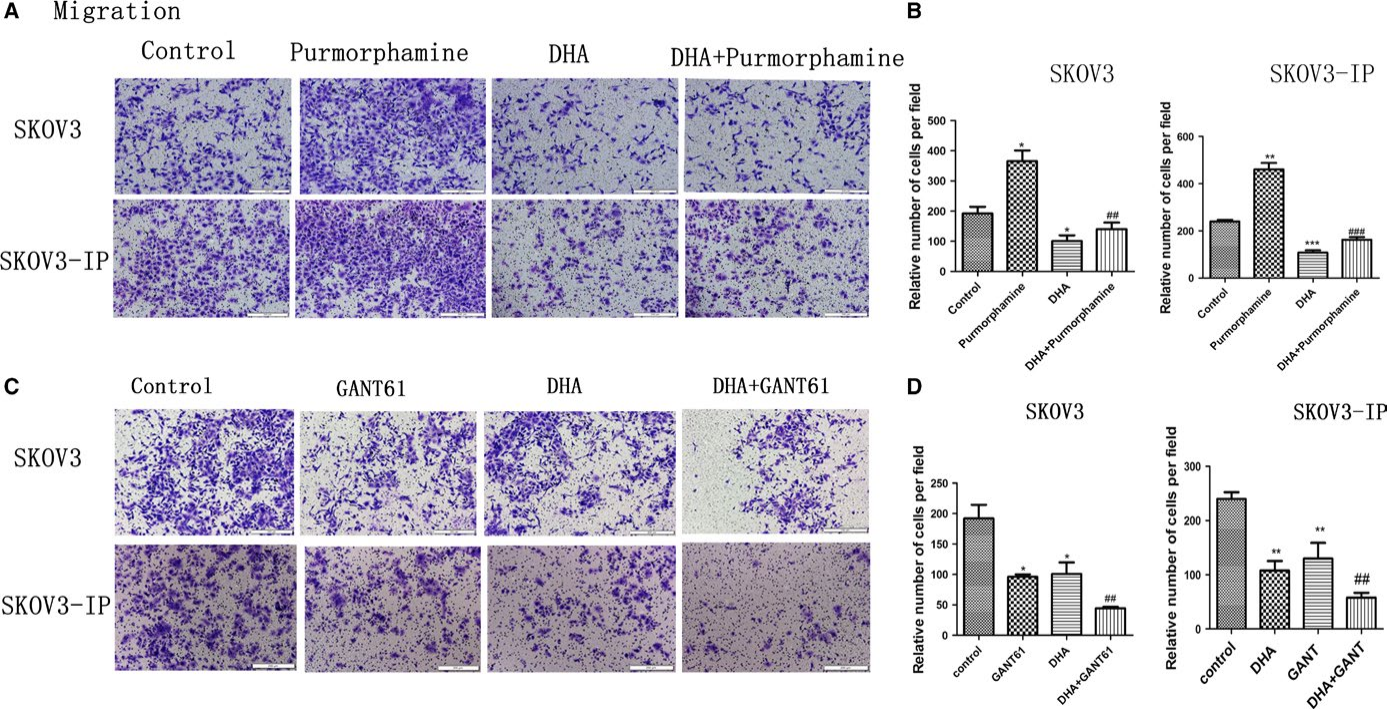
DHA suppresses migration capacity of ovarian cancer through inhibition of the Hh pathway. A, SKOV3 and SKOV3‐IP cells were treated with purmorphamine (1.5 µM) alone, DHA (40 µM) alone, or a combination of DHA and purmorphamine for 24 h. C, SKOV3 and SKOV3‐IP cells were treated with GANT61 (40 µM), DHA (40 µM), or a combination of DHA and GANT61 for 24 h. A and C, Images of migrated cells, which were recorded using an Olympus microscope (10×). B and D, Average number of migrated cells from five randomly selected fields represented by the histogram. Data are represented as the mean ± SEM of three separate experiments. *P < 0.05, **P < 0.01 and ***P < 0.001, compared with controls. ^#^ *P* < 0.05, ^##^ *P* < 0.01 and ^###^ *P* < 0.001, vs the purmorphamine and GANT61 groups

**Figure 8:**
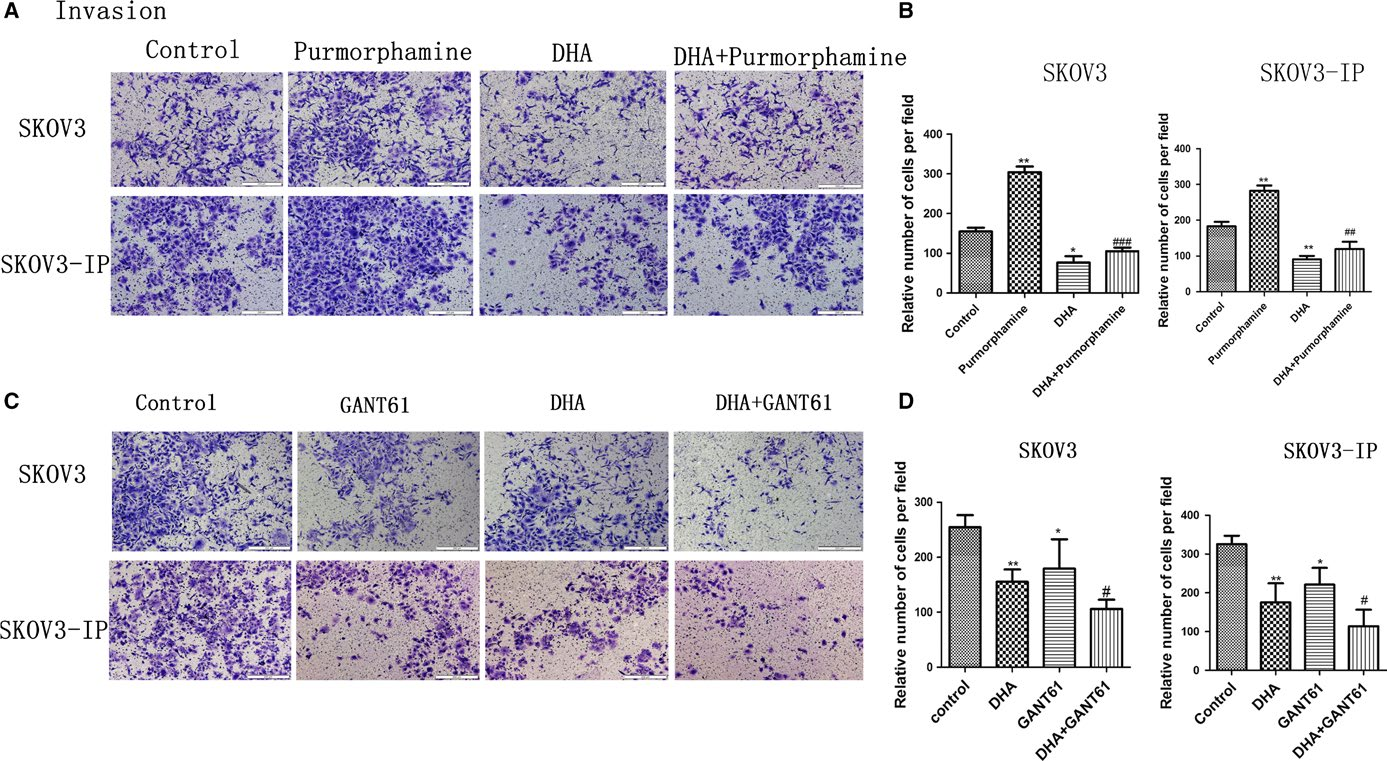
DHA suppresses the invasion capacity of ovarian cancer through inhibition of the Hh pathway. A, SKOV3 and SKOV3‐IP cells were treated with purmorphamine (1.5 µM), DHA (40 µM), or a combination of DHA and purmorphamine for 48 h. C, SKOV3 and SKOV3‐IP cells were treated with GANT61 (40 µM), DHA (40 µM), or a combination of DHA and GANT61 for 48 h. A and C, Images of invaded cells, which were recorded using an Olympus microscope (10×). B and D, Average number of migrated cells from five randomly selected fields was represented by the histogram. Data represent the mean ± SEM of three separate experiments. *P < 0.05, **P < 0.01 and ***P < 0.001, compared with controls. ^#^ *P* < 0.05, ^##^ *P* < 0.01 and ^###^ *P* < 0.001, vs the purmorphamine and GANT61 groups

To further confirm DHA’s antimigration and anti‐invasion effects, we treated SKOV3 and SKOV3‐IP cells with purmorphamine alone (1.5 μM), DHA alone (40 μM), or a combination of DHA and purmorphamine for 24 hours (migration assay) or 48 hours (invasion assay). We also treated SKOV3 and SKOV3‐IP cells with GANT61 alone (40 μM), DHA alone (40 μM), or combination of DHA and GANT61 for 24 hours (migration assay) or 48 hours (invasion assay). We found that 1.5 μM of purmorphamine significantly enhanced migration and invasion ability of SKOV3 and SKOV3‐IP cells (Figures 7A and 8A). Combination of DHA and purmorphamine decreased the effect of purmorphamine‐induced enhancement of cell migration and invasion (Figures 7A and 8A). As demonstrated in Figures 7C and 8C, 40 μM GANT61 significantly decreased migration and invasion of SKOV3 and SKOV3‐IP cells, while cells treated with both DHA and GANT61 displayed significantly decreased migratory and invasive abilities compared to those treated with GANT61 alone. The above results indicate that DHA inhibits purmorphamine‐induced enhancement of cell migration and invasion and augments GANT61’s ability to inhibit migration and invasion in ovarian cancer. Hence, we conclude that DHA‐induced suppression of migration and invasion in ovarian cancer cell may occur via inhibiting the Hh pathway.

## 4 DISCUSSION

Dihydroartemisinin, the primary active product of artemisinin and its derivatives, has been used in malaria treatment for many years. Recently, an increasing number of studies have indicated that DHA has the ability to inhibit tumors, including ovarian cancer,^8^, ^9^, ^10^, ^11^ suggesting that DHA may become a reliable chemotherapeutic agent for the treatment of ovarian cancer. Our research showed that DHA suppresses cell proliferation, cell migration, and cell invasion and induces apoptosis in epithelial ovarian cancer cells. Furthermore, DHA significantly reduced the hedgehog signaling pathway at both mRNA and protein levels. DHA inhibited purmorphamine (smoothened agonist, a specific activator of the Hh signaling pathway)‐induced enhancement of cell proliferation, cell migration, and invasion and the inhibition of apoptosis. DHA augmented GANT61 (Hh pathway inhibitor)‐induced apoptosis and inhibition of cell proliferation, migration, and invasion. Our research suggests that DHA inhibits cell proliferation, cell migration, and invasion and induces apoptosis via suppressing Hh signaling in epithelial ovarian cancer.

First, the effects of DHA on cell proliferation, apoptosis, cell migration, and cell invasion were measured by CCK8 assay, flow cytometry and transwell membrane chambers, respectively. Previous studies reported that DHA suppressed growth and metastasis of HO8910‐PM cells.^11^, ^13^ In our study, DHA significantly inhibited cell proliferation and induced apoptosis in a dose‐dependent manner in epithelial ovarian cancer cells (SKOV3, SKOV3‐IP, HO8910, and HO8910‐PM) in vitro, and DHA has little effect on cell viability and apoptosis in HOSEPICs, in accordance with previous studies.^13^, ^28^ Migration and invasion play a critical role in tumorigenesis and cancer progression. Our research also found that DHA significantly suppressed cell migration and invasion of all ovarian cancer cells. However, a previous study found that DHA did not significantly affect the invasive capacity of SKOV3 cells.^29^ The reason for this discrepancy may be due to different cell states and/or different concentrations of DHA. Our results indicate that DHA inhibits cell proliferation, cell migration, and invasion and induces apoptosis in epithelial ovarian cancer cells.

The Hh pathway is very important for embryonic development;^17^, ^30^ however, many studies suggest that activated Hh pathway promotes tumorigenesis and development of cancer, including ovarian cancer.^25^, ^26^, ^31^, ^32^ The Hh pathway exhibits elevated expression in ovarian cancer.^25^, ^26^, ^33^ Inhibiting hedgehog signaling led to apoptosis and suppression of cell viability, cell migration, and cell invasion in ovarian cancer.^25^, ^26^, ^27^ These studies indicate that inhibition of Hh pathway may serve as a therapeutic target for ovarian cancer. We then investigated the relationship between DHA and the Hh pathway. To examine whether Hh signaling was activated, we assessed protein expression of Hh pathway participants in ovarian cancer cells and HOSEPICs using Western blot. We found that Shh, Ptch1, Smo, and Gli‐1 were overexpressed at the protein level in all ovarian cancer cells compared with HOSEPICs. We also observed that DHA significantly inhibited Smo, Gli‐1, and Ptch1 significantly mRNA and protein expression levels in SKOV3 and SKOV3‐IP cells. However, DHA did not exert significant effects on Shh at the mRNA or protein level. Hence, we speculate that DHA might act on Smo to inhibit Hh signaling, resulting in anticancer effects in ovarian cancer.

To better understand the relationship between DHA and the Hh pathway, SKOV3 and SKOV3‐IP cells were treated with purmorphamine alone, GANT61 alone (Hh pathway inhibitor), DHA alone, combined purmorphamine and DHA, or combined GANT61 and DHA. We found that purmorphamine inhibited apoptosis and enhanced cell proliferation, cell migration, and cell invasion in ovarian cancer cells, while GANT61 induced apoptosis and inhibited cell proliferation, cell migration, and cell invasion in ovarian cancer cells, in accordance with a previous study.^27^ In addition, DHA significantly inhibited the purmorphamine‐induced enhancement of cell proliferation, cell migration, and cell invasion and the inhibition of apoptosis. Furthermore, DHA significantly augmented GANT61‐induced inhibition of cell viability, cell migration, and cell invasion, and increased the level of apoptosis induced by GANT61. These results suggest that DHA may function as an Hh signaling pathway inhibitor by decreasing Smo and GLI1 expression to induce apoptosis and inhibit cell proliferation, migration, and invasion in epithelial ovarian cancer cells.

Taken together, we show for the first time that DHA induces apoptosis and inhibits cell proliferation, migration, and invasion by suppressing Hh signaling. To understand the relationship between DHA and the Hh signaling pathway more thoroughly, additional studies are needed to further explore the mechanism of DHA in ovarian cancer.

## CONFLICT OF INTEREST

All authors declare no conflict of interests.
